# NETO1 Regulates Postsynaptic Kainate Receptors in CA3 Interneurons During Circuit Maturation

**DOI:** 10.1007/s12035-019-1612-4

**Published:** 2019-05-01

**Authors:** Ester Orav, Ilona Dowavic, Johanna Huupponen, Tomi Taira, Sari E. Lauri

**Affiliations:** 1 Molecular and Integrative Biosciences Research Program, University of Helsinki, PO Box 65, Viikinkaari 1, 00014 Helsinki, Finland; 2 HiLife Neuroscience Center, University of Helsinki, Helsinki, Finland; 3 Department of Veterinary Biosciences, University of Helsinki, Helsinki, Finland

**Keywords:** Glutamate receptor, Kainate receptor, Neuropilin tolloid-like protein, Hippocampus, Circuit development, Auxiliary subunit

## Abstract

Kainate type ionotropic glutamate receptors (KARs) are expressed in hippocampal interneurons and regulate interneuron excitability and GABAergic transmission. Neuropilin tolloid-like proteins (NETO1 and NETO2) act as KAR auxiliary subunits; however, their significance for various functions of KARs in GABAergic interneurons is not fully understood. Here we show that NETO1, but not NETO2, is necessary for dendritic delivery of KAR subunits and, consequently, for formation of KAR-containing synapses in cultured GABAergic neurons. Accordingly, electrophysiological analysis of neonatal CA3 *stratum radiatum* interneurons revealed impaired postsynaptic and metabotropic KAR signaling in Neto1 knockouts, while a subpopulation of ionotropic KARs in the somatodendritic compartment remained functional. Loss of NETO1/KAR signaling had no significant effect on development of α-amino-3-hydroxy-5-methyl-4-isoxazolepropionic acid (AMPA) and N-methyl-D-aspartate (NMDA)-receptor-mediated glutamatergic transmission in CA3 interneurons, contrasting the synaptogenic role proposed for KARs in principal cells. Furthermore, loss of NETO1 had no effect on excitability and characteristic spontaneous network bursts in the immature CA3 circuitry. However, we find that NETO1 is critical for kainate-dependent modulation of network bursts and GABAergic transmission in the hippocampus already during the first week of life. Our results provide the first description of NETO1-dependent subcellular targeting of KAR subunits in GABAergic neurons and indicate that endogenous NETO1 is required for formation of KAR-containing synapses in interneurons. Since aberrant KAR-mediated excitability is implicated in certain forms of epilepsy, NETO1 represents a potential therapeutic target for treatment of both adult and early life seizures.

## Introduction

Kainate type ionotropic glutamate receptors (KARs) modulate synaptic transmission and neuronal excitability in various parts of the brain and exhibit subunit, subcellular compartment, and cell type dependent functions [1–3]. Various combinations of GluK1–5 subunits form a functional KAR tetramer that may be complemented with neuropilin tolloid-like proteins (NETO1 and NETO2) that act as KAR auxiliary subunits [1, 3]. NETOs provide an additional level of regulation to KAR localization and function in neurons [4]. For instance, at the glutamatergic CA3-MF synapse in the hippocampus, NETO1 regulates synaptic targeting, biophysical properties, and ligand affinity of postsynaptic GluK2/3-containing KARs [5–7].

KARs are expressed in hippocampal interneurons and, when activated, have robust effects on GABAergic transmission [1–3, 8, 9]. Based on pharmacological studies, genetic mouse models, and mRNA expression pattern, interneuronal KARs in the hippocampus contain subunits GluK1, GluK2, or both [7, 10–15]. Of the GluK1 c-terminal splice variants, GluK1b is specifically expressed in interneurons while GluK1c is restricted to principal cells and mainly expressed during early development [16]. While KARs mediate a prominent inward current in response to agonist application, the synaptic KAR-mediated excitatory postsynaptic current (EPSC), described in CA1 interneurons, is rather modest [10, 17, 18]. Activation of KARs increases interneuron firing by membrane depolarization that consequently leads to higher GABAergic drive onto principal cells. In addition, axonal and presynaptic KARs regulate GABA release, likely via both ionotropic and metabotopic G protein coupled signaling [1–3, 8, 9]. At immature CA3 *stratum lucidum* interneurons, tonically activated KARs have a developmentally restricted G protein coupled function that regulates interneuron excitability by inhibiting medium after hyperpolarizing current *I*_mAHP_ [19]. Thus, hippocampal interneurons contain distinct subpopulations of KARs that regulate interneuron excitability and GABAergic drive both in the immature and in the adult hippocampi.

Numerous studies have documented NETO/KAR functional interactions in the principal neurons in adult [20] and recently also in the newborn hippocampus [21]; however, much less is known about NETO involvement in regulating KARs in interneurons. Neto1 is expressed in GABAergic interneurons in the adult hippocampus [5, 22] where it regulates agonist-induced KAR currents and KAR-dependent recruitment of inhibitory drive onto principal cells [22].

Here, we have further studied the role of NETO1/KAR interaction in hippocampal interneurons, focusing on its physiological role during the first 2 weeks of postnatal development. We show that NETO1, but not NETO2, is necessary for dendritic delivery of KAR subunits and, consequently, for formation of KAR containing synapses in GABAergic neurons. In contrast to principal neurons where KARs promote formation and maturation of glutamatergic synapses [21, 23–26], loss of NETO1/KAR signaling had no significant effects on development of AMPAR-containing synapses in GABAergic interneurons in culture or in area CA3 of the hippocampus. Moreover, NETO1 was not indispensable for maintenance of basal network excitability in the immature hippocampus but was necessary for kainate-induced modulation of GABAergic transmission and network bursts.

## Methods

### Animals

Male and female wild-type (WT), Neto1 knockout (KO), and Neto2KO (C57Bl/6NCrl) mice [6, 27] were used in this study. The animal experiments were performed in accordance with the University of Helsinki Animal Welfare Guidelines.

### Cell Culture and Lentiviral Infection

Primary hippocampal dispersed neuron culture was prepared from P0–2 old WT, Neto1KO, and Neto2KO mice pups as previously described [21]. Cultured neurons were infected with lentiviruses to express GFP, GluK1b-flag, GluK1c-flag, and GluK2-myc at days in vitro 3 (DIV3) and fixed at DIV14 using 4% PFA 4% Sucrose in PBS. Lentiviral plasmids and production of virus particles used in this study were described earlier by Vesikansa and colleagues in 2012 [16].

### Immunofluorescence

Fixed neurons were permeabilized with 0.2% Triton X-100 and incubated with PBS-based blocking solution containing 5% goat serum, 2% bovine serum albumin (BSA), 0.1% Triton X-100, and 0.05% Tween-20. Primary antibodies (Table 1) were diluted in the blocking solution and incubated overnight at + 4 °C. Secondary antibodies (Table 2) were diluted in PBS and incubated 1 h at room temperature.

**Table 1:** Primary antibodies used in this study

Antibody	Dilution	Product nr	Manufacturer
Guinea pig anti-Synaptophysin	1:2000	101,004	Synaptic Systems
Mouse anti-PSD95	1:1000	75–028	NeuroMab
Chicken anti-GAD67	1:2000	198,006	Synaptic Systems
Mouse anti-GAD67	1:1000	MAB5406	Millipore
Rabbit anti-GAD67	1:2000	198,013	Synaptic Systems
Rabbit anti-flag	1:1000	F7425	Sigma Aldrich
Rabbit anti-myc	1:1000	06–549	Millipore
Chicken anti-MAP2	1:8000	AB5543	Millipore
Mouse anti-GluA2/4	1:2500	MAB396	Millipore

**Table 2:** Secondary antibodies used in this study

Antibody	Dilution	Product nr	Manufacturer
Goat anti-chicken Alexa Fluor 405	1:2000	ab175674	Abcam
Goat anti-mouse Alexa Fluor 488	1:2000	A11029	Life Technologies
Goat anti-guinea pig Alexa Fluor 568	1:2000	A11075	Life Technologies
Goat anti-rabbit Alexa Fluor 647	1:2000	A-21245	Molecular Probes
Goat anti-mouse Alexa Fluor 647	1:2000	A21236	Life Technologies

The stained samples were mounted on microscope slides using Prolong Gold antifade reagent (P36934, Life Technologies). Confocal images were acquired using a LSM Zeiss 710 confocal microscope (alpha Plan-Apochromat 63×/1.46 OilKorr M27 objective) for KAR dendritic targeting experiments. All other samples were imaged using Leica TCS SP8 confocal microscope and HC PL APO 93×/1.30 motCORR STED WHITE (glycerol) and 3× digital zoom to obtain high resolution images for synapse analysis.

### Image Analysis

All samples were blinded for genotype and KAR subunit expression during staining, image acquisition, and analysis. Samples included in the analysis were obtained from 2 to 3 independent culture batches. *n* number represents the total number of neurons analyzed. GAD67 staining was used in all samples to identify GAD67+ putative interneurons. Under our culture conditions, 9.7% ± 1.0% of cultured neurons was positive for GAD67 (*n* = 333). Dendritic and axonal targeting of overexpressed tagged-KARs was analyzed in MAP2 positive dendrites and MAP2 negative axons of GAD67+ neurons using SynD/MATLAB [28] by measuring flag or myc intensity along the neurite and normalizing it to soma intensity. Synaptic clusters were visualized using co-staining against presynaptic (Synaptophysin, red) and postsynaptic (PSD95, green) markers. Synaptic cluster density was analyzed from 3D construction using Imaris software. The Synaptophysin (Syn) and PSD95 puncta included in the analysis had spot diameter of at least 0.5 μm on *XY*-axis and 1 μm on *Z*-axis. Syn and PSD95 spots within a distance of 0.7 μm were considered as synaptic clusters. In some samples, KAR and/or GluA2/4-containing synapses were analyzed. Similarly, minimum KAR or GluA2/4 spot diameter on *XY*-axis was 0.5 μm and on *Z*-axis 1 μm. KAR and GluA2/4 spots were considered to co-localize if the distance between the spots were max 0.35 μm. KAR spot was considered to be extrasynaptic if the distance between the flag/myc spot and PSD95 spot was above 0.35 μm. All spot size measurements were calculated from the center of the spot using Imaris software. Syn, PSD95, KAR, and GluA2/4 spots, and synaptic clusters were confirmed visually.

### Acute Slice Preparation

Acute parasagittal hippocampal sections (350 μm) were prepared from brains of WT and Neto1KO mice using dissection solution containing (in mM): 87 NaCl, 2.5 KCl, 7 MgCl_2_, 1.25 NaH_2_PO_4_, 0.5 CaCl_2_, 25 NaHCO_3_, 50 d-sucrose, and 25 d-glucose and equilibrated with 95% O_2_ and 5% CO_2_. Slices were transferred to artificial cerebrospinal fluid (ACSF) containing (in mM): 124 NaCl, 3 KCl, 1.25 NaH_2_PO_4_, 3 MgSO_4_, 26 NaHCO_3_, 2 CaCl_2_, and 15 d-glucose and incubated 30 min at + 35 °C and at 30 min—4 h at room temperature before use.

### Electrophysiological Recordings

Whole-cell voltage clamp recordings were performed from CA3 *stratum radiatum* interneurons that were visually identified with differential interference contrast (DIC) optics. No further identification of interneuron subtype was undertaken.

During the recordings, the chamber was continuously perfused with ACSF (32 °C) bubbled with 95% O_2_ and 5% CO_2_. AMPAR–KAR-mediated responses were recorded in the presence of 100 μM picrotoxin (Abcam) and 50 μM d-AP5 (HelloBio) at a holding potential − 70 mV. NMDAR-mediated responses were recorded at a holding potential + 40 mV and in the presence of 10 μM CNQX (Abcam) and 100 μM picrotoxin. One micromolar of tetrodotoxin (TTX, Abcam) was added to the drug cocktail for recording of miniature excitatory postsynaptic currents (mEPSC). In order to isolate KAR component of the evoked response, AMPAR-selective antagonist GYKI53655 (30 μM) was added to the bath solution. In some experiments, GluK1-specific antagonist ACET (200 nM, Tocris) and agonist ATPA (1 μM, Tocris) were used.

Glutamatergic currents were recorded with 3–5 MΩ glass electrodes filled with Cs-based intracellular solution containing (in mM): 130 CsMeSO_4_, 10 HEPES, 0.5 EGTA, 4 Mg-ATP, 0.3 Na-GTP, 5 QX-314, 8 NaCl, and 285 mOsm (pH 7.2). For evoked EPSC, the stimulation electrode was placed in CA3 *stratum radiatum*. For recording of after hyperpolarizing current (*I*_mAHP_), the filling solution contained (in mM): 130 K-gluconate, 10 HEPES, 10 KCl, 4 ATP-Mg, 0.3 GTP-Mg, 0.2 EGTA, and 285 mOsm (pH 7.2). *I*_mAHP_ was induced by applying depolarizing 60 mV 40 ms step from holding potential of − 47 mV. Spontaneous network activity was recorded from CA3 pyramidal neurons voltage-clamped at − 47 mV with pipette filling solution containing (in mM): 135 K-gluconate, 10 HEPES, 2 KCl, 2 Ca(OH)_2_, 5 EGTA, 4 Mg-ATP, and 0.5 Na-GTP. Spontaneous action potential firing was measured in cell-attached configuration using 10 MΩ electrodes filled with ACSF.

Data were collected using Axoscope 9.2 (Axon instruments) and WinLTP software [29]. For whole-cell patch clamp recordings uncompensated series resistance (*R*_s_ < 30 MΩ) was monitored, and cells were discarded if *R*_s_ varied more than 20%.

Spontaneous events were analyzed with MiniAnalysis 6.0.3 program (Synaptosoft Inc.) and calculated in 1 min bins. Events were verified visually, and events with amplitude less than three times the baseline noise level were rejected. Evoked EPSC and *I*_mAHP_ amplitude was analyzed using WinLTP. Holding current data were collected during *I*_mAHP_ recordings.

### Statistical Analysis

All statistical analysis was performed on raw data using SigmaPlot software. First, the data distribution was tested with Shapiro–Wilk test. Then, one-way ANOVA with Holm–Sidak post hoc comparison or Kruskal–Wallis test was used accordingly as stated. Student’s paired *t* test was used to assess the treatment effect as indicated. All data are presented as mean ± SEM; *p* < 0.05 was considered statistically significant. In figures, the significance levels are indicated by asterisks as follows: *p < 0.05, **p < 0.01, and ***p < 0.001.

## Results

### NETO1 Regulates Dendritic Targeting but Not Synaptic Recruitment of KAR Subunits GluK1b and GluK2 in GAD67+ GABAergic Neurons In Vitro

NETO1/2 has been suggested to promote plasma membrane entry and synaptic targeting of KAR subunits depending on the cell type and KAR subunit identity [4, 20]. However, currently, there is no consensus on the precise mechanisms that guide the subcellular compartmentalization of KARs at GABAergic interneurons. Therefore, we characterized the subcellular localization of recombinant tagged KAR subunits in cultured GAD67 positive neurons from wild-type (WT), NETO1- and NETO2-deficient mice, focusing on the subunits GluK1 and GluK2. Both the GluK1b and GluK1c splice variants were included in the analysis.

**Fig 1:**
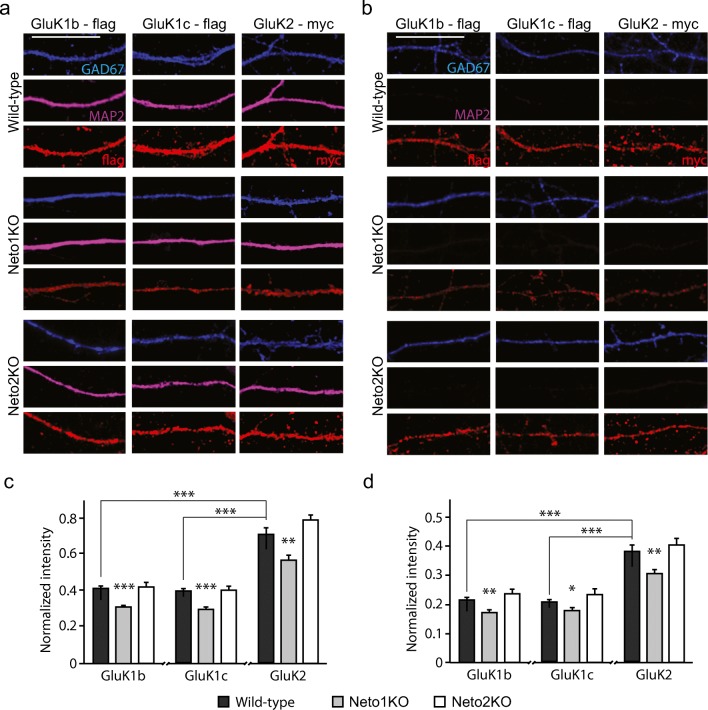
NETO1 regulates dendritic and axonal delivery of KAR subunits in cultured GAD67+ GABAergic neurons. **a** Example images depicting the localization of overexpressed GluK1b-flag, GluK1c-flag and GluK2-myc (red) in dendrites of cultured WT, Neto1KO and Neto2KO GABAergic neurons. MAP2 (purple) is used as dendritic marker, while GAD67 staining (blue) identifies GABAergic neurons. Scale bar 20 μm. **b** Examples illustrating the localization of overexpressed GluK1b-flag, GluK1c-flag, and GluK2-myc (red) in MAP2-negative axons of GABAergic neurons in the same cultures as in **a**. Scale bar 20 μm. **c** Quantification of the dendritic targeting of KAR subunits GluK1b (*n* = 49, *n* = 34, *n* = 18), GluK1c (*n* = 49, *n* = 33, *n* = 15), and GluK2 (*n* = 42, *n* = 32, *n* = 18) in WT (black bar), Neto1KO (gray bar), and Neto2KO (white bar) GAD67+ neurons, respectively. **d** Quantification of the axonal targeting of KAR subunits GluK1b (*n* = 40, *n* = 34, *n* = 12), GluK1c (*n* = 45, *n* = 33, *n* = 12), and GluK2 (*n* = 23, *n* = 26, *n* = 18) in WT (black bar), Neto1KO (gray bar), and Neto2KO (white bar) GAD67+ neurons, respectively. For quantification, GluK signal intensity in the neurites is normalized to the soma intensity. *p < 0.05; **p < 0.01; ***p < 0.001

All the GluK subunits studied were targeted to MAP2 positive dendrites and MAP2 negative axons in WT GAD67+ neurons. The relative intensity of GluK2 was higher as compared to GluK1b and GluK1c in both dendrites (GluK2 0.71 ± 0.04; GluK1b 0.41 ± 0.01, *p* < 0.001, Kruskal–Wallis; and GluK1c 0.40 ± 0.01, *p* < 0.001, Kruskal–Wallis) and axons (GluK2 0.38 ± 0.02; GluK1b 0.21 ± 0.01, *p* < 0.001, Kruskal–Wallis; and GluK1c 0.21 ± 0.01, *p* < 0.001, Kruskal–Wallis) of GAD67+ neurons. Dendritic delivery of all three subunits was significantly lower in the dendrites of GAD67+ Neto1KO neurons as compared to controls (GluK1b 75.3% ± 2.2%, *p* < 0.001, Kruskal–Wallis; GluK1c 74.6% ± 2.6%, *p* < 0.001, Kruskal–Wallis; GluK2 79.9% ± 3.5%, *p* = 0.01, Kruskal–Wallis) (Fig. 1a, c). Also, axonal delivery of the KAR subunits was significantly lower in the GAD67+ Neto1KO neurons than controls (GluK1b 80.6% ± 3.80%, *p* = 0.001; GluK1c 86.0% ± 4.1%, *p* = 0.031, Kruskal–Wallis; GluK2 79.7% ± 4.0%, *p* = 0.004) (Fig. 1b, d). In contrast, we found no differences in dendritic (GluK1b 101.6% ± 6.2%, *p* = 0.82; GluK1c 101.3% ± 4.6%, *p* = 0.69; GluK2 109.5% ± 3.4%, *p* = 0.072) (Fig. 1a, c) or axonal (GluK1b 110.0% ± 7.5%, *p* = 0.26; GluK1c 112.3% ± 8.5%, *p* = 0.11; GluK2 105.7% ± 5.9%, *p* = 0.49) (Fig. 1b, d) delivery of the tested KAR subunits in Neto2KO neurons as compared to WT controls. Therefore, we excluded Neto2KO from further study.

**Fig. 2:**
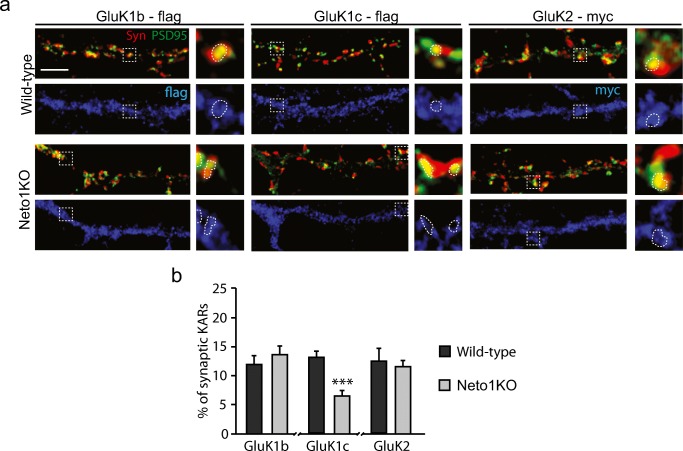
Synaptic localization of KAR subunits in cultured WT and Neto1KO GABAergic neurons. **a** Example images of immunostaining against Synaptophysin (Syn, red), PSD95 (green), and tagged KAR subunits (flag/myc, blue) in dendrites of WT and Neto1KO GAD67+ neurons. Enlarged insets of a single synapse cluster (co-localization of Syn and PSD95, circled with a dashed line) and corresponding synaptically and extrasynaptically located GluK1b-flag, GluK1c-flag, and GluK2-myc clusters. GAD67 staining not shown for clarity. Scale bar 20 μm. **b** Pooled data showing percent of synaptically located KARs in WT (black; GluK1b-flag *n* = 16, GluK1c-flag *n* = 17, GluK2-myc *n* = 20) and Neto1KO (gray; GluK1b-flag *n* = 20, GluK1c-flag *n* = 10, GluK2-myc *n* = 20) GAD67+ neurons. Scale bar 20 μm. *p < 0.05; **p < 0.01; ***p < 0.001

We then analyzed the distribution of the KAR subunits between synaptic and extrasynaptic pools, using puncta with co-localized Synaptophysin (Syn) and PSD95 staining as a synapse marker. At WT GAD67+ neurons, only a minority of the recombinant GluK subunits co-localized with Syn-PSD95 positive puncta (GluK1b 12.0% ± 1.4%, GluK1c 13.1% ± 1.0%, GluK2 12.5% ± 2.2%). This distribution was not significantly altered in Neto1KO for the subunits GluK1b and GluK2 (GluK1b 13.6% ± 1.4%; GluK2 11.5% ± 1.2%). However, proportion of synaptically located GluK1c in Neto1KO GABAergic neurons (6.4% ± 1.1%) was significantly lower as compared to WTs (*p* < 0.001) (Fig. 2a, b).

Thus, under our culture conditions, subcellular localization of KAR subunits in GABAergic neurons depends on endogenous expression of NETO1 but not NETO2. Our findings further suggest that NETO1 regulates dendritic targeting rather than synaptic recruitment of interneuron specific KAR subunits GluK1b and GluK2*.* In contrast, synaptic localization of the GluK1c splice variant, which is endogenously expressed in the hippocampal pyramidal neurons during early development [16], was dependent on NETO1 expression.

### NETO1 Is Required for Postsynaptic and Metabotropic KAR Functions in Immature CA3 Interneurons

Interneurons in area CA3 of hippocampus express NETO1 already during the first week of life [21]. To understand the physiological functions of NETO1/KAR complex in the immature GABAergic neurons, we performed electrophysiological recordings in CA3 *stratum radiatum* interneurons in acute hippocampal slices from neonatal WT and Neto1KO mice.

**Fig. 3:**
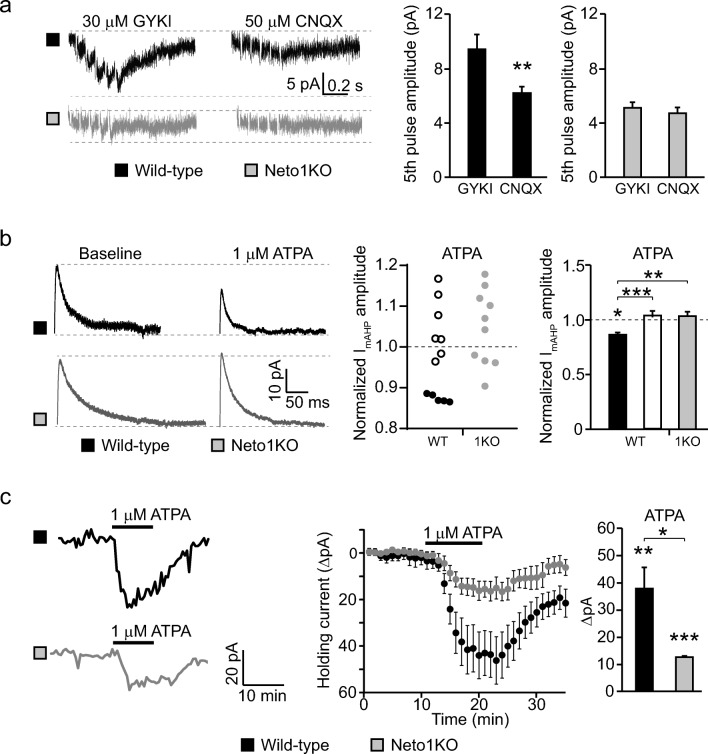
NETO1 regulates postsynaptic and metabotropic KAR functions in neonatal CA3 *stratum radiatum* interneurons. **a** Example traces and pooled data demonstrating the effect of 30 μM GYKI and 50 μM CNQX on EPSC, evoked by 5 pulse at 50 Hz stimulation of mixed afferents, in WT (*n* = 10) and Neto1KO (*n* = 7) CA3 *stratum radiatum* interneurons at P4–6. **b** Example traces and quantified data illustrating the effect of ATPA (1 μM) on *I*_mAHP_ currents. Pooled data show the effect of ATPA on *I*_mAHP_ in WT cells, where ATPA decreased *I*_mAHP_ amplitude over 10% (black, *n* = 6), the remaining WT cells (white, *n* = 8), and Neto1KO interneurons (grey, *n* = 10) at P4–6. **c** Example traces and pooled data showing the effect of ATPA (1 μM) on holding current during voltage clamp recordings in CA3 *stratum radiatum* interneurons in WT (*n* = 12) and Neto1KO (*n* = 10) at P4–6. *p < 0.05; **p < 0.01; ***p < 0.001

Postsynaptic KARs contribute to synaptic transmission at certain interneurons [10, 17, 30]; however, it is not known whether KAR-mediated EPSCs at GABAergic neurons are modulated by NETO1. To investigate this, we placed a stimulation electrode to CA3 *stratum radiatum* in order to activate all possible glutamatergic inputs to the recorded cell and used pharmacological tools to isolate putative KAR-mediated postsynaptic currents. At P5 WT slices, application of the AMPAR-selective antagonist GYKI53655 (30 μM) in the presence of antagonists for NMDAR and GABA_A_ receptors revealed a small slowly decaying EPSC in response to 5 pulse 50 Hz afferent stimulation (peak amplitude 9.4 ± 1.1 pA) (Fig. 3a). This current component is likely mediated by synaptic KARs as it was significantly reduced with AMPAR–KAR antagonist CNQX (50 μM; EPSC amplitude to 65.5% ± 4.8% of control, *p* = 0.001, Student’s *t* test) (Fig. 3a).

Having detected functional synaptic KARs in immature CA3 interneurons in WTs, we then performed the same experiment using Neto1KO slices. Application of GYKI53655 completely blocked the EPSCs in response to 5 pulse 50 Hz stimulation in Neto1KO slices (peak amplitude 5.1 ± 0.4 pA), and application of CNQX had no further effect on the response (92.8% ± 7.5% of control) (Fig. 3a). These data indicate that ionotropic KARs are present at the glutamatergic synapses inCA3 *stratum radiatum* interneurons, where they mediate a modest EPSC that is not observed in the absence of NETO1.

In addition to the ionotropic function, KARs in CA3 *stratum lucidum* interneurons activate a G protein coupled signaling pathway that inhibits medium after hyperpolarizing potassium current (*I*_mAHP_) during the first week of postnatal development [19]. To investigate the possible role of NETO1 in the metabotropic signaling initiated by GluK1-containing KARs, we studied the effect of GluK1-selective agonist ATPA (1 μM) on *I*_mAHP_ in neonatal (P4–6) WT and Neto1KO slices. The *I*_mAHP_ amplitude was not different between the genotypes under basal conditions (WT 137.0 ± 19.8 pA, Neto1KO 139.3 ± 14.5 pA). ATPA had no significant effect on WT *I*_mAHP_ currents when all recorded cells were included in the analysis (97.7% ± 3.5% of control; *n* = 12). However, ATPA significantly decreased *I*_mAHP_ amplitude in 5 out of the 12 WT cells (87.4% ± 0.4% of control, *p* = 0.03, Student’s *t* test). In contrast, ATPA did not reduce *I*_mAHP_ in any of the 10 recorded CA3 interneurons in Neto1KO (104.7% ± 2.9% of control) (Fig. 3b). It should be noted that our recordings included interneurons located in CA3 *stratum radiatum*, while the previous study was restricted to interneurons in CA3 *stratum lucidum* [19], probably explaining why the effect of ATPA was observed only in a subpopulation of the neurons.

During these recordings, ATPA induced an inward current in WT interneurons, detected as an increase in the holding current during the whole-cell voltage clamp recording (Δ*I* 39.3 ± 8.9 pA). Interestingly, a clear ATPA-induced current was also detected in Neto1KO interneurons (Δ*I* 12.3 ± 2.1 pA); however, it was significantly smaller as compared to WT (*p* = 0.007, Kruskal–Wallis) (Fig. 3c).

Taken together, these findings show that NETO1 is required for many, but not all, of the functions described for KARs in immature GABAergic interneurons. Loss of NETO1 impaired postsynaptic and metabotropic KAR signaling, while a subpopulation of ionotropic GluK1-containing KARs remained functional.

### Absence of NETO1 Selectively Impairs Formation of KAR-Containing Synapses in GAD67+ GABAergic neurons

Both ionotropic and metabotropic KAR signaling have been implicated in synaptogenesis and synapse maturation in hippocampal principal neurons [16, 23, 31]. Therefore, we next investigated the role of NETO1/KAR complex in the formation of glutamatergic synapses in cultured GAD67+ neurons from WT and Neto1-null mice.

**Fig. 4:**
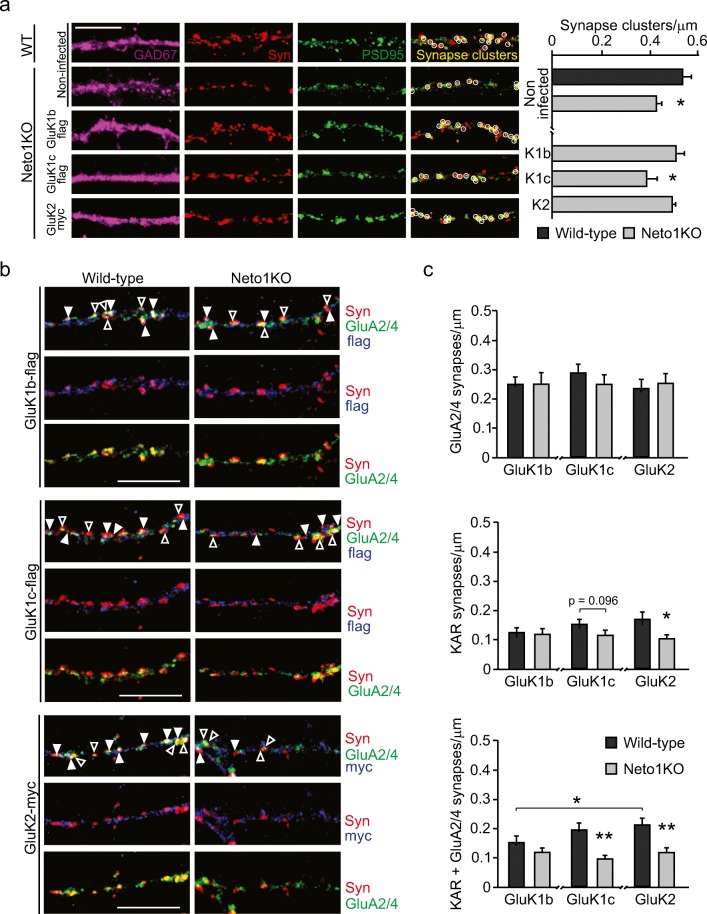
Loss of NETO1 leads to selective impairment of KAR-containing synapses in cultured GAD67+ GABAergic neurons. **a** Example images showing immunostaining against GAD67 (purple), Synaptophysin (Syn, red) and PSD95 (green) in hippocampal neurons. Merged images show synapses (co-localization of Syn and PSD95, circled) in non-infected WT (*n* = 35) and Neto1KO (*n* = 30), and in GluK1b-flag (*n* = 20), GluK1c-flag (*n* = 10) and GluK2-myc (*n* = 20) overexpressing Neto1KO GAD67+ neurons. Graphs show the quantified synapse density for the corresponding experimental conditions. Scale bar 10 μm. **b** Example images of Synaptophysin (Syn, red); GluA2/4 (green); and GluK1b-flag, GluK1c-flag, or GluK2-myc (blue) staining in WT and Neto1KO GAD67+ neurons. Synapses are identified as having a contact with Synaptophysin and highlighted with arrowheads: GluA2/4 positive (open arrowhead), KAR positive (closed arrowhead), or KAR and GluA2/4 positive (closed arrowhead). GAD67+ staining not shown for clarity. Scale bar 10 μm. **c** Quantified data for the density of GluA2/4 positive, KAR positive, or KAR and GluA2/4 positive synapses in WT and Neto1KO GAD67+ neurons. GluK1b-flag (WT *n* = 20, Neto1KO *n* = 20), GluK1c-flag (WT *n* = 20, Neto1KO *n* = 18), or GluK2-myc (WT *n* = 19, Neto1KO *n* = 20). *p < 0.05; **p < 0.01; ***p < 0.001

The density of synapses, identified as puncta with co-localized PSD95 and Synaptophysin (Syn) staining, was significantly lower in Neto1KO GAD67+ neurons (0.42 ± 0.02/μm) as compared to WT (0.53 ± 0.03/μm, *p* = 0.01) (Fig. 4a). This phenotype was KAR-dependent as it was fully rescued with the overexpression of either GluK1b (0.51 ± 0.04/μm) or GluK2 (0.49 ± 0.01/μm), but not GluK1c (0.39 ± 0.04/μm, *p* = 0.04, as compared to WT) in Neto1KO cultures (Fig. 4a).

To further characterize this phenotype, we analyzed the density of KAR and AMPAR-containing synapses in KAR-overexpressing GAD67+ neurons. AMPAR synapses were defined as co-localized clusters of staining against endogenous GluA2/4 and Syn and represented 44% of the glutamatergic synapses in the WT GAD67+ neurons. KAR synapses were visualized as clusters of overexpressed tagged KAR subunits that co-localized with Syn (25% of synapses). The remaining 31% of glutamatergic synapses contained both AMPARs (GluA2/4) and KARs.

The density of synapses that contained only AMPARs in GAD67+ neurons was not different between WT and Neto1KO cultures, irrespective of the overexpressed KAR subunit (GluK1b 0.25 ± 0.02/μm, 0.25 ± 0.04/μm; GluK1c 0.29 ± 0.03/μm; GluK2 0.23 ± 0.03/μm, 0.25 ± 0.03/μm; WT and Neto1KO, respectively) (Fig. 4b, c; open arrowhead). In contrast, the density of KAR-only synapses was significantly more sparse in Neto1KO as compared to WTs in GluK2-expressing (0.17 ± 0.02/μm and 0.10 ± 0.01/μm, *p* = 0.02, Kruskal–Wallis, WT and Neto1KO, respectively), but not in GluK1-expressing GAD67+ GABAergicneurons (GluK1b 0.12 ± 0.02/μm and 0.12 ± 0.02/μm; GluK1c 0.15 ± 0.02/μm and 0.11 ± 0.02/μm, WT and Neto1KO, respectively) (Fig. 4b, c; closed arrowhead). Similarly, the density of synapses containing both KARs and AMPARs was affected by loss of NETO1 as evidenced by lower density of GluK1c + GluA2/4 or GluK2 + GluA2/4-containing synapses in Neto1KO as compared to WT controls (GluK1c 0.19 ± 0.02/μm and 0.09 ± 0.01/μm, *p* = 0.001, Kruskal–Wallis; GluK2 0.21 ± 0.02/μm and 0.11 ± 0.02/μm, *p* = 0.02, Kruskal–Wallis, WT and Neto1KO, respectively) (Fig. 4b, c; closed arrowhead).

Taken together, these data suggest that loss of NETO1 selectively impairs the formation of KAR-containing synapses in GABAergic neurons while AMPAR synapses appear to differentiate normally. The loss of KAR-containing synapses in the Neto1KO cultures can fully explain the lower density of glutamatergic synapses, identified by PSD95-Syn co-localization in GAD67+ neurons (Fig. 4a).

### Loss of NETO1 Has No Effect on AMPAR and NMDAR-Mediated Synaptic Inputs in CA3 Interneurons

We went on to analyze whether absence of NETO1 affected functional glutamatergic input in CA3 *stratum radiatum* interneurons by recording spontaneous action potential independent miniature EPSC (mEPSC) at two different stages of development (P5 and P15). Both pharmacologically isolated AMPAR–KAR-mediated mEPSCs (mEPSC_AMPA-KA_) and NMDAR-mediated mEPSC (mEPSC_NMDA_) were studied using hippocampal slices from WT and Neto1KO mice.

**Fig. 5:**
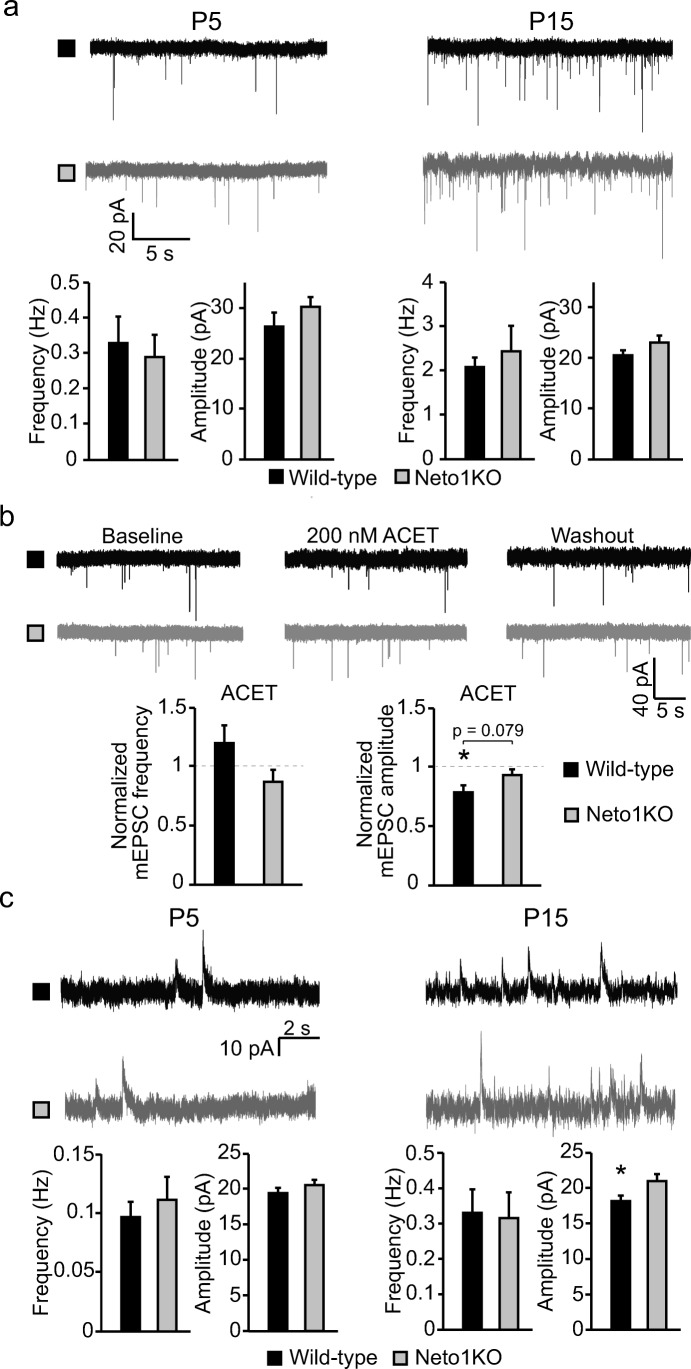
NETO1 deficiency has no significant effect on the development of AMPAR- or NMDAR-mediated synaptic input into CA3 *stratum radiatum* interneurons. **a** Example traces and pooled data illustrating AMPAR-mediated mEPSCs recorded from CA3 *stratum radiatum* interneurons in WT (black, *n* = 12, *n* = 17) and Neto1KO (gray, *n* = 14, *n* = 15) slices from P4–6 to P14–16 mice, respectively. **b** Example traces and quantified data illustrating the effect of GluK1-selective antagonist ACET (200 nM) on mEPSC frequency and amplitude in WT (*n* = 8) and Neto1KO (*n* = 6) P4–6 CA3 *stratum radiatum* interneurons. **c** Example traces and pooled data depicting NMDAR-mediated mEPSCs recorded from CA3 *stratum radiatum* interneurons in WT (black, *n* = 13, *n* = 14) and Neto1KO (gray, *n* = 9, *n* = 13) slices from P4–6 and P14–16 mice, respectively. *p < 0.05; **p < 0.01; ***p < 0.001

There was no significant difference between the genotypes in mEPSC_AMPA-KA_ at either developmental stage (frequency, P5 0.33 ± 0.07 Hz and 0.29 ± 0.06 Hz; P15 2.07 ± 0.22 Hz and 2.42 ± 0.57 Hz; amplitude P5 26.3 ± 2.7 pA and 30.2 ± 1.9 pA; P15 20.5 ± 1.0 pA and 23.1 ± 1.3 pA; for WT and Neto1KO, respectively) (Fig. 5a). Interestingly, application of the GluK1-selective agonist ACET (200 nM) significantly reduced mEPSC_AMPA-KA_ amplitude in P5 WT (79.0% ± 5.8% of control, *p* = 0.04, Student’s *t* test) but not in Neto1KO interneurons (93.7% ± 4.3% of control) (Fig. 5b), supporting that immature CA3 interneurons contain NETO1-dependent synaptic KARs that have a minor contribution to postsynaptic current. In these experiments, no significant effect of ACET on the frequency of mEPSC_AMPA-KA_ was detected in either genotype (119.7% ± 15.1% and 87.3% ± 9.9%, for WT and Neto1KO, respectively).

Similar to mEPSC_AMPA-KA_, we detected no differences between the genotypes in mEPSC_NMDA_ at P5 (frequency 0.10 ± 0.01 Hz and 0.11 ± 0.02 Hz, amplitude 19.4 ± 0.8 pA and 20.5 ± 0.8 pA, for WT and Neto1KO, respectively). At P15, however, the mEPSC_NMDA_ amplitude was slightly higher in Neto1KO interneurons (WT 18.1 ± 0.8 pA, Neto1KO 20.8 ± 1.0 pA, *p* = 0.04) while there was no difference between the genotypes in their frequency (WT 0.33 ± 0.07 Hz, Neto1KO 0.31 ± 0.07 Hz) (Fig. 5c).

NETO1 has been shown to be an interaction partner of NMDA receptors [7, 27] and to regulate the subunit composition of NMDAR at MF-CA3 synapses [7]. Therefore, we further tested the possibility that NETO1 affects NMDAR subunit composition in immature interneurons (P4–6). Application of ifenprodil (5 μM), an antagonist selective for the NR2B-containing receptors, reduced the amplitude of evoked NMDAR-mediated EPSCs both in WT (63.8% ± 16.7%, *n* = 5) and Neto1KO slices (74.8% ± 11.1%, *n* = 9), but this effect was not significantly different between the genotypes (data not shown). Therefore, we concluded that NETO1 does not affect synaptic NMDARs in CA3 interneurons during early postnatal development.

Taken together, these data show that loss of NETO1 has no major effects on AMPAR- and NMDAR-mediated synaptic transmission in CA3 interneurons.

### Loss of NETO1 Has Minor or No Effects on the Excitability of Immature Hippocampal Network

**Fig. 6:**
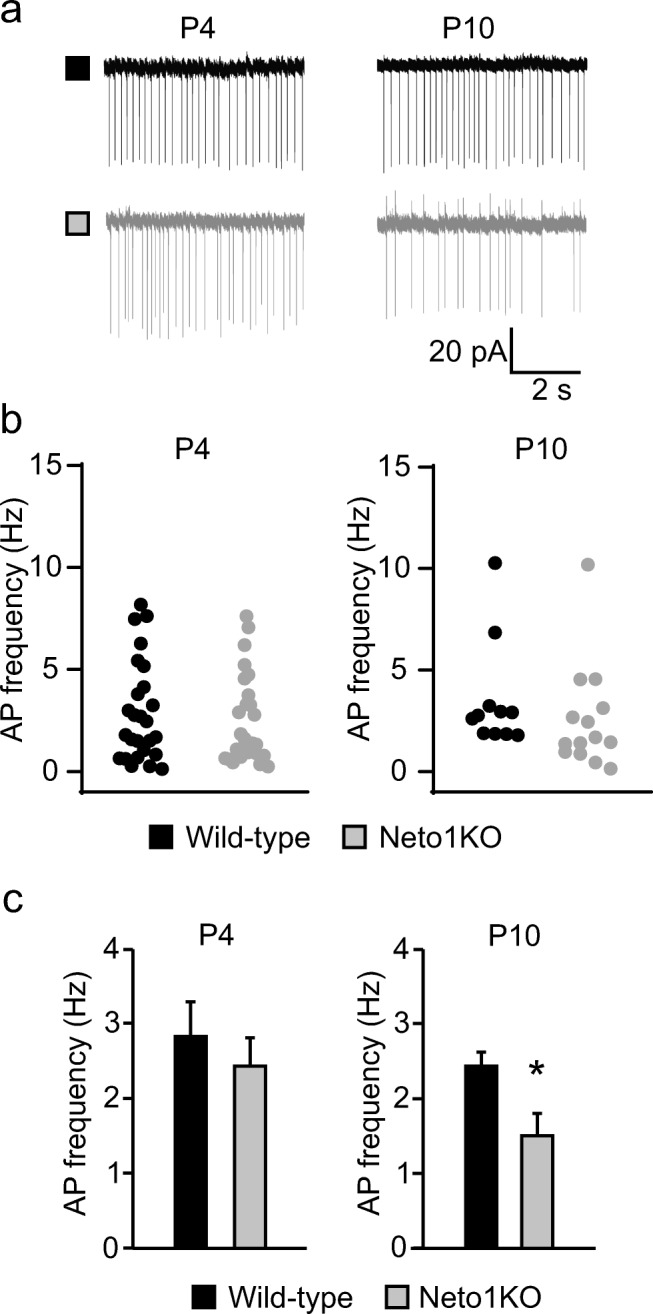
Spontaneous action potential firing of CA3 *stratum radiatum* interneurons in WT and Neto1KO slices. **a** Example traces of cell-attached recordings illustrating spontaneous action potential firing of CA3 *stratum radiatum* interneurons of WT (black) and Neto1KO (gray) slices at P4 and P10. **b** Graphs showing the mean action potential firing frequency of individual CA3 *stratum radiatum* interneurons at P3–6 (*n* = 18, *n* = 17) and P9–10 (*n* = 12, *n* = 14) in WT and Neto1KO slices, respectively. **c** Pooled data of mean action potential firing frequency of CA3 *stratum radiatum* interneurons at P3–6 (*n* = 18, *n* = 17) and P9–10 (*n* = 9, *n* = 11) from WT and Neto1KO, respectively. Cells with high firing frequency are excluded from the P9-10 age group. *p < 0.05; **p < 0.01; ***p < 0.001

Hippocampal KARs have been shown to modulate early network activity [19, 32, 33], characterized by spontaneously occurring network bursts [34] that are highly dependent on intact excitation–inhibition balance [35]. To evaluate the significance of NETO1 in regulation of network excitability in the immature hippocampus, we first recorded spontaneous action potential (AP) firing of CA3 *stratum radiatum* interneurons using cell-attached recordings from P3 and P10 WT and Neto1KO mice (Fig. 6a). At P3–6, we observed a large heterogeneity in the firing frequency of individual recorded cells in both WT and Neto1KO, but no significant differences between genotypes (mean AP frequency, WT 2.82 ± 0.46 Hz; Neto1KO 2.42 ± 0.38 Hz) (Fig. 6b, c). The interneuron subtypes become more differentiated by P10 with some cells acquiring distinctive high action potential firing frequency. The mean AP firing frequency was not different between the genotypes at P10 when all cells were included in the analysis (3.27 ± 0.78 Hz, *n* = 12; 2.56 ± 0.69 Hz, *n* = 14; WT and Neto1KO, respectively). However, if the cells with high (> 5 Hz) firing frequency were excluded from the analysis, we observed a significantly slower spontaneous firing of Neto1KO interneurons (1.51 ± 0.28 Hz, *p* = 0.02) as compared to WT (2.43 ± 0.19 Hz) at P10 (Fig. 6b, c).

**Fig. 7:**
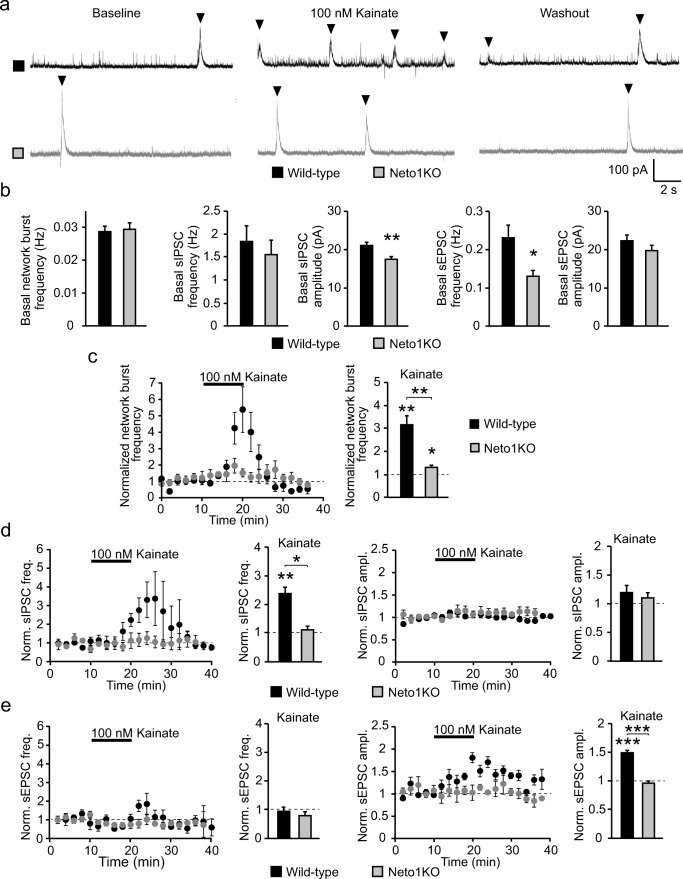
NETO1/KAR complex increases the kainate sensitivity of the immature CA3 network. **a** Example traces illustrating the effect of 100 nM kainate (KA) on spontaneous network activity recorded from P5 CA3 pyramidal neurons in WT and Neto1KO slices. With the use of low chloride electrode filling solution, GABAergic and glutamatergic synaptic events are seen as outward and inward currents, respectively. The network bursts are indicated with black arrowheads. **b** Pooled data illustrating the frequency of network bursts (*n* = 29, *n* = 30), sIPSCs (*n* = 14, *n* = 14), and sEPSCs (*n* = 14, *n* = 14) as well as the amplitude of sIPSCs and sEPSC in P4–6 CA3 principal neurons in WT (black) and Neto1KO (gray) slices, respectively. **c** Time course plot and pooled data demonstrating the effect of 100 nM kainate on network bursts in CA3 pyramidal neurons of P4–6 WT (*n* = 7) and Neto1KO (*n* = 5) mice. **d** Time course plots and pooled data showing effect of 100 nM kainate on sIPSC frequency and amplitude in CA3 pyramidal neurons of P4–6 WT (*n* = 6) and Neto1KO (*n* = 3) mice. **e** Corresponding data for sEPSCs (WT, *n* = 7; Neto1KO, *n* = 4). *p < 0.05; **p < 0.01; ***p < 0.001

To further study the role of NETO1 in neonatal network activity, we recorded spontaneous synaptic currents from WT and Neto1KO CA3 pyramidal cells that receive GABAergic inputs from CA3 interneurons. By using a low chloride concentration in the pipette filling solution, we were able to analyze the frequency of network bursts, spontaneous inhibitory (sIPSC) and spontaneous excitatory postsynaptic currents (sEPSC) from the same recordings. The basal frequency of network bursts was not different between the genotypes at P4–6 (WT 0.032 ± 0.003 Hz; Neto1KO 0.029 ± 0.002 Hz) (Fig. 7a, b). Consistent with the data showing no difference in the interneuron firing frequency, the frequency of sIPSCs in the CA3 pyramidal neurons was similar in WT and Neto1KO slices (1.46 ± 0.84 Hz and 1.28 ± 0.16 Hz, respectively). However, sIPSC amplitude was lower in Neto1KO (17.1 ± 0.6 pA) as compared to WT (21.2 ± 1.0 pA, *p* = 0.005) (Fig. 7b). Lastly, sEPSC frequency, but not amplitude, was significantly lower in Neto1KO CA3 pyramidal cells as compared to WT (frequency 0.23 ± 0.03 Hz and 0.13 ± 0.01 Hz, *p* = 0.04; amplitude 22.3 ± 1.7 pA and 19.7 ± 1.2 pA, for WT and Neto1KO, respectively) (Fig. 7b).

Thus, NETO1 deficiency had minor or no effects on the spontaneous network activity in area CA3 during the first week of life. Despite its strong effects on interneuronal KARs, NETO1 is not indispensable for physiological network activity, and the immature circuit lacking NETO1 is able to maintain the excitability similar to that in the WT.

### NETO1-Deficient Network Is Less Sensitive to Kainate

Since the excitability of the neonatal hippocampal network is under strong homeostatic control [36], mechanisms that modulate circuit activity might be compensated for in a knockout model and thus not observed under basal conditions. Therefore, we went on to investigate whether the NETO1-deficient network was able to respond to low concentration of kainate (KA), which potently increases excitability of the hippocampal network already during the first postnatal week [33].

As expected, application of 100 nM KA resulted in a robust increase in the occurrence of network bursts in CA3 pyramidal neurons in WT slices (P4–6) (313.6% ± 39.7% of baseline, *p* = 0.001, Student’s *t* test) (Fig. 7a, c). In the Neto1KO slices, 100 nM KA induced a small increase in the frequency of network bursts (129.3% ± 9.0% of baseline, *p* = 0.02, Student’s *t* test) (Fig. 7a, c). However, KA effect on the burst frequency in WT was significantly larger as compared to Neto1KO (*p* = 0.003, Kruskal–Wallis) (Fig. 7a, c).

In addition to the increase in the network bursts, 100 nM KA caused a large increase in the frequency of sIPSCs (237.6% ± 41.7% of baseline, *p* = 0.007, Student’s *t* test) in WT slices but had no significant effect on sIPSC amplitude (119.6% ± 9.1% of baseline) (Fig. 7d). Consistent with the loss of somatodendritic ionotropic KARs in interneurons, KA had no effect on sIPSCs in Neto1KO slices (frequency 110.9% ± 37.2% of baseline; amplitude 110.2% ± 8.9% of baseline) (Fig. 7d). Analysis of spontaneous glutamatergic currents indicated an increase in sEPSC amplitude in response to KA application in WT (150.1% ± 6.0% of baseline, *p* < 0.001, Student’s *t* test) but not in Neto1KO slices (94.7% ± 5.3% of baseline; *p* < 0.001 between genotypes) (Fig. 7e). KA had no effect on sEPSC frequency either in WT (94.0% ± 14.0% of baseline) or Neto1KO (79.8% ± 12.9% of baseline) (Fig. 7e).

These data provide evidence that NETO1 is critical for KA-induced network bursts and participates in recruiting the inhibitory drive from immature GABAergic interneurons to CA3 pyramidal cells.

## Discussion

KARs have developmentally restricted functions in the hippocampus both in principal cells [21, 26, 31] and in interneurons [19, 37]. We have previously shown that NETO1 is critical for the immature-type KAR functions and maturation of the connectivity between CA3-CA1 principal neurons [21], while the role of NETOs at GABAergic interneurons in the neonatal hippocampus is not previously characterized. In the present study, we provide the first description of NETO1-dependent subcellular targeting of KAR subunits in GABAergic interneurons. Furthermore, we show that NETO1 regulates both ionotropic and metabotropic KAR functions in CA3 interneurons already during the first week of life and is critical for KA-induced modulation of network bursts and GABAergic transmission at the immature network.

### NETO1 and Subcellular Targeting of KARs at GABAergic Neurons

NETO1 is expressed in interneurons in the neonatal [21] and adult hippocampus [5, 22], where it is co-expressed with KAR subunits GluK1, 2, and 5 in different interneuron subpopulations [22]. While the role of NETO1 in targeting of various KAR subunits has been studied in glutamatergic neurons [5–7, 21, 38–40], no previous data from GABAergic neurons exists.

Our data show that a predominant effect of NETO1 on interneuronal KARs is to promote their dendritic and axonal targeting, which was significantly impaired in NETO1-deficient GABAergic neurons irrespective of the KAR subunit identity. We recently reported a similar role for NETO1 in axons of hippocampal principal neurons [21]. In contrast to NETO1, NETO2 appears to not be involved in axonal and dendritic delivery of interneuronal KAR subunits, at least under our culture conditions.

In the principal neurons, NETO1 is also reported to promote the postsynaptic capture of GluK1 and GluK2 [6, 7] or selectively GluK1 [39, 40]. Here, we observed no difference in synaptic recruitment of KAR subunits GluK1b and GluK2 between WT and NETO1-deficient interneurons. In contrast, the synaptic distribution of GluK1c subunit, which is endogenously mainly expressed in principal neurons of the immature hippocampus, was significantly impaired in the Neto1KO interneurons. These data suggest that NETO1 has subunit and cell-type specific effects on KAR trafficking at the level of synapses [38, 40], while promoting distal targeting of KARs in a subunit independent manner in both glutamatergic and GABAergic neurons.

### NETO1/KAR-Dependent Synaptic Signaling in Immature Interneurons

Functional characterization of immature CA3 interneurons indicated that both ionotropic and metabotropic KAR signaling were compromised or completely lost in the absence of NETO1, similar to that previously shown for CA3 principal neurons in adult hippocampus [5–7]. Thus, we identified a small KAR-mediated component of EPSC in the WT but not Neto1KO interneurons in response to 50 Hz/5 pulse afferent stimulation. Also, the G protein coupled regulation of *I*_mAHP_ via GluK1 subunit containing KARs was not observed in NETO1-deficient interneurons. Intriguingly, however, agonist application revealed a subpopulation of functional ionotropic GluK1-containing KARs in NETO1-deficient CA3 *stratum radiatum* interneurons supporting the idea that NETO1 has distinct effects on different types of KARs even within one neuron.

Postsynaptic KAR-mediated current has been previously described in adult CA3 pyramidal cells [41, 42] and in CA1 interneurons [10, 17]. In addition, there is evidence suggesting that KARs and AMPARs in CA1 interneurons are located at distinct synapse populations [15]. Interestingly, in CA1 pyramidal cells, NETO1 overexpression targets KARs preferentially to synapses that contain no AMPARs [39], providing a plausible mechanism for generation of KAR-containing AMPAR-lacking synapses in GABAergic interneurons where NETO1 is endogenously expressed. Indeed, analysis of AMPAR- and KAR-containing synaptic clusters in cultured GABAergic neurons identified synapses that contained only AMPAR, only KAR, but also synapses with both AMPARs and KARs. In the absence of NETO1, the density of both KAR-only and AMPAR–KAR synapses were reduced, while AMPAR-containing synapses were not affected. It should be noted that these data were obtained using overexpression of recombinant KAR subunits in cell culture, which might override some endogenous targeting mechanisms. However, also functional analysis supported existence of NETO1-dependent postsynaptic KARs in immature CA3 interneurons. Therefore, the most likely interpretation of these results is that ionotropic NETO1/KAR signaling operates in a small fraction of synapses in the CA3 interneurons during early postnatal development and exhibits a rather modest contribution to postsynaptic current.

### NETO1 Regulates Formation of KAR-Containing Synapses but Has No Effect on Maturation of AMPAR- and NMDAR-Mediated Transmission in Interneurons

KARs have been implicated in synaptogenesis and synaptic maturation in the CA1 circuitry [21, 24, 26, 31] as well as in the mossy fiber-CA3 synapse [23, 25]. In addition, KARs regulate maturation of the dendritic tree in both, principal neurons and interneurons [43, 44]. Our data from cell cultures showed that loss of NETO1 is associated with compromised synaptogenesis that can be rescued with overexpression of KARs in GABAergic neurons. However, this effect could be fully explained by the loss of KAR-containing synapses, while AMPAR-containing synapses formed apparently normally in the absence of NETO1.

The rescue of KAR-synapses in NETO1-deficient GABAergic neurons was subunit dependent. GluK1c overexpression was not able to rescue the impaired synapse formation in the Neto1KO, most likely because it is not efficiently recruited to postsynaptic compartments in GABAergic Neto1KO neurons. However, overexpression of GluK1b and GluK2 subunits rescued the overall density of synapses in the Neto1KO GABAergic neurons to the WT level. Since NETO1 had no effect on the synaptic distribution of GluK1b and GluK2 in the GABAergic neurons, the loss of KAR-containing synapses in the Neto1KO is likely due to the impaired dendritic delivery of KAR subunits which limits the number of available receptors at the synaptic site. In support to this idea, overexpression of GluK2 subunit in the WT was associated with a significantly higher density of KAR-containing synapses as compared to GluK1b, which is not delivered to dendrites as efficiently as GluK2.

Consistent with the lack of effect of NETO1 on synaptic AMPAR clusters in cultured interneurons, we found no significant differences in the AMPAR-mediated transmission to WT vs Neto1KO interneurons in the area CA3 of neonatal mice. Interestingly, NMDAR transmission exhibited NETO1-dependent phenotype by the end of the second postnatal week. Therefore, we tested for the possibility that NETO1 regulates the NMDAR subunit composition in the interneurons, similar to that shown for CA3 principal cells [7], which could consequently affect development of glutamatergic inputs to interneurons [45]. However, no difference in the ifenprodil sensitivity of NMDAR-mediated current was detected between the genotypes.

Thus, while our data identify a role for NETO1 in promoting formation of KAR-containing synapses, lack of NETO1/KAR signaling had no apparent consequences on development and maturation of AMPAR–NMDAR-mediated synaptic transmission in GABAergic interneurons. Together with previous data [21, 23–26, 31], these results support that KARs regulate development of glutamatergic transmission in a cell type specific manner, promoting maturation of AMPAR-mediated transmission in principal glutamatergic neurons, but not in GABAergic interneurons. Further, our data suggest that KAR-synapses at GABAergic interneurons are not developmental precursors of AMPA-containing synapses but rather represent a distinct population of synapses, whose physiological significance at the immature circuitry remains elusive.

### NETO1/KAR Complex Has Little or No Effect on the Excitability of the Immature Hippocampal Network but Is Required for KA Modulation

KARs are potent regulators of interneuron excitability in the neonatal hippocampus [19, 32, 37]. However, we observed no effect of NETO1 on the basal spontaneous action potential firing of CA3 interneurons during the first week of postnatal development, in contrast to significantly lower firing of GluK1KO interneurons at the same developmental stage [19]. Consistently, under basal conditions, NETO1 deficiency had no effect on the medium after hyperpolarizing current, proposed to underlie the differences AP firing frequency in neonatal GluK1-lacking interneurons [19]. The excitability of the neonatal network is under strict homeostatic control [36, 46–48]; thus, it is feasible that any NETO1-dependent mechanisms regulating action potential firing frequency are compensated for and thus not manifested in a knockout model.

Interestingly, during the second postnatal week and in parallel with maturation of interneuronal firing properties, the spontaneous AP firing frequency became lower in NETO1-deficient CA3 interneurons as compared to WT controls. Possibly, at the mature network, postsynaptic NETO1/KAR signaling controls temporal summation of EPSCs and thereby contributes to spike generation in CA3 interneurons, similar to that shown for CA3 pyramidal neurons [5, 49] and CA1 interneurons [18]. Alternatively, the altered firing frequency in the Neto1KO interneurons could reflect some developmental delay in their maturation [44].

In accordance with the unchanged interneuron firing during the first postnatal week, immature-type spontaneous network activity, recorded from CA3 pyramidal neurons, was not markedly affected by the absence of NETO1. The main observed effect, i.e., a significant reduction in the frequency of sEPSCs, is likely due to loss of presynaptic KARs that tonically facilitate glutamate release at this developmental stage [21, 31, 32]. However, similar to that shown previously in adult Neto1KO [22], the immature NETO1-deficient CA3 network was strikingly less sensitive to KA modulation. Application of 100 nM KA had only modest effects in the Neto1KO in contrast to the robust induction of network bursts in the WT [33]. KA activates KARs in various subcellular compartments in the pyramidal neurons and interneurons, and both cell types express NETO1 in the neonate CA3. Our data do not resolve which KAR population is mainly responsible for the loss of KA-dependent network bursts in the Neto1KO. However, previous data have identified that low concentration of KA induce ectopic spiking of CA3 pyramidal neurons that initiates network bursts in the neonatal hippocampus [33], suggesting that loss of axonal KARs in Neto1KO CA3 pyramidal neurons [21] contributes to this phenotype.

KA application in the CA3 network also associates with a large increase in the frequency of spontaneous GABAergic events, an effect that has been attributed to KAR-mediated depolarization of GABAergic neurons and their axons as well as regulation of GABA release [1–3, 8, 9]. This effect is strongly impaired in adult NETO1-deficient mice [22], indicating a central role for NETO1 in recruitment of GABAergic inputs to CA3 pyramidal neurons in response to KA application. Consistent with these previous findings, we found that the effect of 100 nM KA on sIPSCs in the immature CA3 network was lost in Neto1KOs. Interestingly, axonal delivery of KAR subunits was significantly impaired in cultured Neto1KO GABAergic neurons, suggesting that loss of axonal and presynaptic KARs, together with the deficit in somatodendritic KARs, contributes to the low KA sensitivity of GABAergic transmission in the Neto1KO. In support to this idea, it was recently reported that NETO1 is required for presynaptic KAR function at CCK/CB1 interneurons in the adult hippocampus [22]. However, we cannot rule out the possibility that reduced affinity of KARs in the absence of NETO1 also contributes to the observed phenotype [5, 50, 51].

Taken together, these findings support that while NETO1 is required for many of the functions ascribed to KARs in immature CA3 interneurons, NETO1 deficiency does not have severe consequences on the basal excitability of the CA3 network during early postnatal development. However, NETO1 is central for KA-dependent modulation of the network activity and GABAergic synaptic transmission already during the first week of life. Given that aberrant KAR-mediated transmission has been implicated in certain forms of epilepsy [52], NETO1 might provide an attractive target for development of novel treatments against adult and early life seizures.
